# Reimagining Researchers in Health Research Comment on "Experience of Health Leadership in Partnering With University-Based Researchers in Canada: A Call to ‘Re-Imagine’ Research"

**DOI:** 10.15171/ijhpm.2020.05

**Published:** 2020-01-20

**Authors:** Katrina M. Plamondon

**Affiliations:** School of Nursing, Faculty of Health and Social Development, University of British Columbia, Kelowna, BC, Canada.

**Keywords:** Researcher Roles, Research Relationships, Knowledge Translation, Humility

## Abstract

It is widely accepted that research evidence should inform policy and practice in health service organizations. Yet, amid increasingly complex and even wicked realities, where health inequities prevail and resource-strained health service organizations struggle to keep pace with demand, using research to inform practice and policy remains an elusive ideal. Bowen and colleagues’ study illuminates critical relational pathways for engagement in evidence-informed practice and decision-making and suggests beginning insights into what might contribute to the tenuousness of this aspirational ideal. But what *kind of* reimagination is needed to move toward more genuine engagement in research? This commentary argues for reimagining the *relationship between researchers and health research* , positioning researchers as responsive, guided by humility, and part of a greater collective effort to advance a public good. It challenges notions of objectivity and detached expertise, suggesting that researchers embrace an active practice of* humility* focused on approaching research in service and from a position of learning rather than knowing.

## Introduction

Research evidence is widely recognized as essential for informing policy and practice in health service organizations—an integral contributor to effectiveness and quality in healthcare design and delivery.^1,2^ Yet, amid increasingly complex and even wicked realities, where health inequities prevail and resource-strained health service organizations struggle to keep pace with demand, *using* research to inform practice and policy remains an elusive ideal^3^ with significant lags between the generation and application of knowledge.^4^ Through their research with health service organization managers, Bowen and colleagues’^5^ study illuminates critical pathways for engagement in evidence-informed practice and decision-making and offers beginning insights into what might contribute to the tenuousness of this aspirational ideal. But what *kind* of reimagination is needed to move toward more genuine engagement in research?

Building on Bowen and colleagues’ analysis, and more than a decade of my own experience serving as a bridge between academia and health service organizations, I concur that the *relationship between researchers and health research* is among what warrants reimagining. In particular, there is room to re-imagine research relationships as responsive, guided by humility (an intentional commitment to approaching research from a position of learning rather than knowing), and part of a greater collective effort to advance a public good.

Bowen andcolleagues’ study demonstrates how inextricable relationships are from evidence-informed policy and practice, pointing to the influence of the postures, attitudes, and approaches researchers adopt when working in partnership. A demonstration of this inextricability in their findings was health service organization managers’ perceptions of the low value of research and researchers in their work. Bowen et al describe health service organization managers’ experiences of research as often “unhelpful or irrelevant” (p. 1), with study participants connecting the attitudes and responsiveness of individual researchers—and what they are exposed to during their careers—as a key determinant of how useful their contributions may be. Managers in health service organizations carry complex workloads, ripe with demands for producing ‘data’ that demonstrates performance, quality, and efficiency. They are often obligated to perform and report on programmatic and outcome indicators as a functional requirement to show how the system is (or is not) meeting expectations.^6,7^ Pressure to report under tight timelines, with great specificity, is intense and obligatory for people in these leadership roles.^8^ If research and researchers are going to play a role in any part of this knowledge generation, it needs to be in a way that reflects a deep understanding of the contextual constraints people in these managerial roles face.

As others have argued, Bowen and colleagues’ study suggests research is infrequently considered an integral or obligatory part of health service organization ‘work.’^9^ Instead, it is often dismissed as irrelevant or elitist, while evaluation is considered requisite and QI as a key to innovation, efficiency, and responsiveness to patient-driven concerns.^5,10,11^ Certainly, there are different purposes and scopes for each; however, they share a common interest in systematically generating new insights and often rely on similar methods for generating and documenting new knowledge. Research could play a valuable role in this work, bringing people with advanced skills to these processes; yet Bowen and colleagues’ findings suggest the potential is lost, at least in part because of a perception that research activities do not fit well within the practical, applied framework of QI.^5^

This perception of impracticality says something not about the relevance and need for systematic learning-from-doing, but the margins between complex worlds and bureaucracies that function with different rules and valued outputs.^12,13^ Indeed, there is no reason that QI cannot emerge from research or evaluation, or evaluation integrated in research, or research from evaluation. Bridging examples continue to emerge, with research funding agencies increasingly interested in incentivizing academics to partner better and do more applied research.^14,15^ Knowledge brokers can act as figurative bridges, making accessible the languages of these different worlds in ways that make their contributions more visible and compelling.^16,17^ But mediating roles, however important for serving as that bridge, can also enable the maintenance of a status quo in both places. Researchers’ roles and relationships, directly, need reimagination.

The health service organization managers contributing to Bowen and colleagues’ study were situated across Canada, in different kinds of health service organizations; yet, most of them shared a sentiment that universities act as though separate from them and society, without appreciation for the day-to-day complexities they juggle. Academics and researchers are perceived as elite and detached. And the perception is not without legitimacy. Researchers are often socialized into detachment as though it represents some form of ‘objectivity’ that strengthens their methodological integrity.^18,19^ Far too often, and particularly for biomedical and bench sciences, researchers are trained with a self-reinforcing obliviousness to the philosophical and power assumptions inherent in their approaches.^20-23^ Taught that their way of knowing is the only way, as a medium of truth beyond critical reprisal, they are actively mentored to avoid advocacy—as though offering their findings alongside a statement of implications or position somehow dilutes the quality of their findings. But this is a false sense of integrity, hiding behind specious standards of ‘objectivity.’^24,25^ Such postures erode the possibility of researchers bringing valuable and applied contributions to the fast-paced, complex world of health service organizations.

These barriers point to the need for different research training and reward approaches that both recognize the value of responsive research relationships and open possibilities for humility. Imagine if, instead of pursuing standards of excellence defined by a patriarchal (and perhaps anachronistic) Academy,^26-28^ researchers invested themselves in creating responsive, service-oriented *relationships* within the organizations where they are working? Rather than tenure and promotion reviews that focused on short-term academic outputs, researchers could be evaluated on the value, strength, and impact of long-term partnerships. Doing so also would invite consideration of a quite different construct of who the researcher is in relationship to health research and to health service organizations, with *humility* a core standard of practice for researchers. Humility can be taught and cultivated as a life-long practice. It requires regular attention and integration in the day-to-day activities and *ways* in which we interact with the vast array of others who are all integral to enabling the process of evidence-informed practice and decision-making.

Adopting a practice of humility, as a researcher, involves “thinking and acting for the right reasons (ie, other-oriented motivations)” (p. 225), involving aspirations for modesty, an ability to evoke empathy during conflict, and an openness to others (cultures, ways of being, worldviews).^29^ It is, in essence, the practice of taking an intentional stance of learning rather than knowing.^30,31^ It is intimately related to understanding the complex socio-political and historical contexts that shape systems and structures in society,^32^ and contribute to the wickedness of persistent health problems. It invites active examination of one’s assumptions and biases,^32^ which can be challenging for those of us working in the health sciences where the privileging of biomedicalism and positivism^33,34^ creates an environment of self-reinforcing tendencies to *not* examine assumptions.

Daring to practice humility in our research, and with those with whom we are partnering, invites a complete reimagination of the relationship between researcher and health research. Rather than positioning ‘researcher as expert,’ researchers are positioned as skilled learner, listener, and responder. They engage in vulnerable examination of the influence of personal, professional, and research values^32,35^ in shaping how and what possibilities are visible at any given moment. They embrace being questioned without defensiveness, and an openness to questioning how the methods they know and can bring to a team can be in service. Importantly, adopting a practice of humility challenges notions of ‘knowing’ with certainty such that researchers foster research relationships grounded in exploration, curiosity, openness to doubt, and questioning what is known and how a team can have confidence about that knowing.^35^ This kind of positioning shapes different possibilities for how research problems are understood and research questions conceptualized.

Researchers, positioned in this way, would strive to collectively create strategies to respond to the daily, lived challenges within health service organizations (and perhaps within society, more broadly). Why does this matter? Because without humility-driven relationships between researcher and health research, there will always be a presumption of knowing better—in terms of shaping how a research problem is come to be identified; is understood, named, or described; and how research questions and studies evolve from these places. Reimagining the relationships between researcher and research positions them not as paternalistic ‘others’ with elite knowledge, but as allies and collaborators with a specific skill set—working together in a collective of people, all of whom bring something critical to a problem-solving intention. Combined with continued efforts to support people working within health service organizations to understand the value and embrace the use of evidence in practice and policy, this shift would serve to align researchers’ and health service managers’ shared goals of improving health and care for the populations they serve.

Together, the perceived value of research and its potential contribution—and the relationship between researcher and health service organizations—speak to another larger reimagination needed. Billions of public dollars are invested in research annually.^36,37^ Research is a promising and important contributor to finding and testing solutions to the pressing problems facing humanity. Perhaps now, more than ever, researchers face an ethical quandary that challenges previously held sanctitude of curiosity-driven research: inherently global health issues, such as the climate crisis, posing challenges demand the full attention of capable minds and hands.^38-40^ Amid broad pushes for ensuring greater impact and return-on-investment, research can be more relevant and responsive when the process of identifying and refining research questions is inclusive and invested in examining values and assumptions^41^—across all domains of research, from clinical trials to those approaches more overtly aligned with social justice orientations.

Research can and should be playing a role in seeking solutions to these pressing challenges. Engaged scholarship, for example, imagines a relationship between researchers, research and society that positions it as part of a greater public good, and therefore obliges responsiveness to pressing social issues with accountability and equity.^42,43^ Indigenous approaches to knowledge translation push further still, situating research and its application as an embodied connection between knowing *as* doing—and as part of reclamation and decolonization.^44,45^ Both offer important points for reflection on the imaginative possibilities for research.As humanity faces the greatest obstacles in recorded history, health service organizations will continue to feel as though collapsing under the weight of demand. Day-to-day, and even moment-to-moment survival keeps those working within these systems preoccupied with the most basic navigation of this demand.

Bowen et al^5^ offer a resonant description of the reasons why engagement in research and knowledge translation reside more in the imaginary than the applied. Their findings ring true to me and others whose experiences as champions for research and knowledge translation have come to the edges of calling for a complete reimagination of the role of research in society.^46,47^ Academic institutions need to embrace a process of reimagining the value *assessed* to service-driven and responsive research, including finding pathways to recognize the importance of time spent building trust and generating products that meet the needs of research *users* (more than tenure and promotion review committees). In alignment with others’ practical wisdom for new and established researchers to cultivate their attention to reflexivity,^33^ power and privilege,^48^ and meaningful, responsive partnerships,^15,49^ Bowen and colleagues’ discussion opens possibilities for transformative consideration of research in society. Indeed, the reimagination I describe invokes a restructuring of tenure and promotion criteria in ways that embrace and value this relational work. Along with protecting intellectual property and publishing in spaces that ‘count,’ authenticity and responsiveness of research in partnership should be valued equally. I wholeheartedly agree for the call to re-imagine research—and extend it. We (researchers, knowledge brokers, health service organization leaders, universities, the public) need to collectively re-imagine the role of research *in society*—with researchers acting from a position of humility to collectively cultivate a public good that can be leveraged to find, test and apply solutions, and create more promising pathways forward.

## Ethical issues

Not applicable.

## Competing interests

Author declares that she has no competing interests.

## Author’s contribution

KMP is the single author of the paper.
